# Transcriptomic Features of Bovine Blastocysts Derived by Somatic Cell Nuclear Transfer

**DOI:** 10.1534/g3.115.020016

**Published:** 2015-09-03

**Authors:** Byungkuk Min, Sunwha Cho, Jung Sun Park, Yun-Gyeong Lee, Namshin Kim, Yong-Kook Kang

**Affiliations:** * Development and Differentiation Research Center, KRIBB, 125 Gwahak‐ro, Yuseong‐gu, Daejeon, 305‐806, South Korea; § Korean Bioinformation Center, KRIBB, 125 Gwahak‐ro, Yuseong‐gu, Daejeon, 305‐806, South Korea; † Department of Functional Genomics, University of Science and Technology (UST), 217 Gajeong‐ro, Yuseong‐gu, Daejeon, 305‐350, South Korea; ‡ Department of Bioinformatics, University of Science and Technology (UST), 217 Gajeong‐ro Yuseong‐gu, Daejeon, 305‐350, South Korea

**Keywords:** RNA-seq, SCNT, transcriptomes, bovine blastocyst, development

## Abstract

Reprogramming incompletely occurs in most somatic cell nuclear transfer (SCNT) embryos, which results in misregulation of developmentally important genes and subsequent embryonic malfunction and lethality. Here we examined transcriptome profiles in single bovine blastocysts derived by *in vitro* fertilization (IVF) and SCNT. Different types of donor cells, cumulus cell and ear-skin fibroblast, were used to derive cSCNT and fSCNT blastocysts, respectively. SCNT blastocysts expressed 13,606 genes on average, similar to IVF (13,542). Correlation analysis found that both cSCNT and fSCNT blastocyst groups had transcriptomic features distinctive from the IVF group, with the cSCNT transcriptomes closer to the IVF ones than the fSCNT. Gene expression analysis identified 56 underrepresented and 78 overrepresented differentially expressed genes in both SCNT groups. A 400-kb locus harboring zinc-finger protein family genes in chromosome 18 were found coordinately down-regulated in fSCNT blastocysts, showing a feature of reprogramming-resistant regions. Probing into different categories of genes important for blastocyst development revealed that genes involved in trophectoderm development frequently were underrepresented, and those encoding epigenetic modifiers tended to be overrepresented in SCNT blastocysts. Our effort to identify reprogramming-resistant, differentially expressed genes can help map reprogramming error-prone loci onto the genome and elucidate how to handle the stochastic events of reprogramming to improve cloning efficiency.

Incomplete reprogramming in somatic cell nuclear transfer (SCNT) embryos causes growth arrest and death at various developmental stages, producing diverse, anomalous phenotypes (Eggan *et al.* 2001; Hill *et al.* 1999. 2000; Lanza *et al.* 2000; Ono *et al.* 2001). Reprogramming errors perturb genetic programming, yielding faulty gene expression profiles, and accumulation of these errors hampers normal development of SCNT embryos (Humpherys *et al.* 2001). Diverse gene expression studies in SCNT embryos have been conducted in bovine by the use of qualitative and quantitative approaches. Most early studies focused on identification of marker genes with the use of reverse transcriptase–polymerase chain reaction (PCR) that would predict developmental competence of cloned embryos derived by various protocols (Amarnath *et al.* 2007; Daniels *et al.* 2000; Donnison and Pfeffer 2004; Li *et al.* 2005, 2006; Wrenzycki *et al.* 2001). However, these studies have produced limited data pertaining to post-SCNT gene expression changes.

The study of preimplantation development can be facilitated by large-scale genomic approaches, but both the scarcity of materials and insufficient technology have hampered full exploitation of such methodologies. In the last decade, however, significant technical progress has been made. Several studies used microarray analysis to analyze whole transcript profiles in bovine SCNT embryos (Aston *et al.* 2009; Beyhan *et al.* 2007; Pfister-Genskow *et al.* 2005; Somers *et al.* 2006). Expression microarrays, however, depend on existing genome annotations, which are disadvantageous when domestic animals, such as cows, are used, whose genome annotations are incomplete. Recent gene expression studies have used high-throughput RNA-sequencing technology (RNA-seq), which allowed surveys of transcriptomes at an unprecedented resolution.

RNA-seq overcomes the main drawbacks of expression microarrays because it can detect unannotated transcriptional activity (without any previous knowledge of the genomes being studied) and distinguish different transcriptional and splicing variants (Huang and Khatib 2010; Wang *et al.* 2009). Therefore, RNA-seq is currently the most popular choice for transcriptomic studies, and the robustness of RNA-seq is highly advantageous for transcriptomic studies in early mammalian embryos. The first RNA-seq study with bovine embryos was performed by Huang and Khatib (2010), where transcriptomes between bovine blastocysts and degenerative embryos were compared. Driver *et al.* (2012) reported transcriptomic difference between *in vivo*− and *in vitro*−derived bovine blastocysts. Unlike the other studies that used pooled embryo samples, Chitwood *et al.* (2013) reported transcriptomes of single *in vitro*−derived bovine blastocysts and characterized transcript sequence variation for allele-specific expression. Until now, unfortunately, RNA-seq−mediated transcriptomic profiling analysis of bovine SCNT embryos has not been reported, which would be no doubt invaluable for gaining deeper insight into the molecular mechanism of reprogramming.

SCNT is a very powerful technique to produce genetically modified animals that can be used as bioreactors of industrial usage and model animals for biomedical research (Niemann and Lucas-Hahn 2012; Zhou *et al.* 2015). However, low cloning efficiency has been limiting the promising applications. The main goal of this study is to survey the transcriptomic landscapes of cloned embryos to provide insights into genomic reprogramming processes after SCNT. We here report RNA-seq transcriptome data from individual bovine blastocysts in three different groups: an *in vitro* fertilization (IVF)-derived blastocyst group and two SCNT groups derived from different donor cells. We compared their transcriptomic data to characterize expression variation among individual blastocysts in each group, between IVF and SCNT groups, and between the two different SCNT groups. To the best of our knowledge, the present study is the first to evaluate bovine SCNT blastocyst features using the RNA-seq transcriptomic approach, which we hope it can help elucidate the mechanism that governs local and global reprogramming events.

## Materials and Methods

### IVF of bovine oocytes

This study was carried out in strict accordance with the recommendations in the Guide for the Care and Use of Laboratory Animals of the National Livestock Research Institute of Korea. The protocol was approved by the Committee on the Ethics of Animal Experiments of the Korea Research Institute of Bioscience and Biotechnology.

We obtained permission from the slaughterhouse (Daejon-Ojung SH, Korea) to use the ovaries. Cumulus−oocyte complexes were obtained from follicles and incubated in *in vitro* maturation medium under paraffin oil for 20 hr at 38.5° in an atmosphere of 5% CO_2_. The medium for oocytes maturation was TCM-199 (Invitrogen) supplemented with 10% (v/v) fetal bovine serum (FBS; Invitrogen), 10 µg/mL FSH-P (Folltropin-V, Vetrepharm), 0.6 mM cysteine, 0.2 mM sodium pyruvate, and 1 µg/mL estradiol-17β. *In vitro*−matured oocytes were fertilized with frozen-thawed sperm at a concentration of 2 × 10^6^/mL in fertilization medium (Park *et al.* 2007). Sperm and oocytes were coincubated at 38.5° in 5% CO_2_ in air. After 20–22 hr of insemination, cumulus-enclosed oocytes were stripped by gentle pipetting and then cultured in CR1aa supplemented with 3 mg/mL bovine serum albumin (fatty acid free). After culture for 3 d, cleaved embryos were further cultured in each well of a four-well culture plate containing 750 mL of CR1aa (with 10% FBS) for 4 d at 38.5° in 5% CO_2_ in air (Koo *et al.* 2002). After 7−8 d of culture, blastocyst formation was observed.

### Somatic cell nuclear transfer

For bovine SCNT blastocysts, we manipulated bovine mature oocytes as we described elsewhere (Koo *et al.* 2002). Oocyte manipulations such as enucleation and cell injection were performed with a micromanipulator equipped with an inverted microscope (Leitz, Ernst Leitz Wetzlar GmbH). The medium used for manipulation was TL-Hepes containing 7.5 μg/mL cytochalasin B. The first polar body and partial cytoplasm presumptively containing metaphase II chromosomes were removed together by the use of a micropipette. Single cells were individually transferred to the perivitelline space of the recipient cytoplast, and the resulting donor-oocyte complexes were equilibrated in a 50-μL drop of cell fusion medium for 10–20 sec and then transferred to a fusion medium of 0.3 M mannitol, 0.5 mM Hepes, 0.01% bovine serum albumin, 0.1 mM CaCl_2_, and 0.1 mM MgCl_2_. The donor−oocyte complexes were induced to fuse with a single pulse of direct current of 1.6 kV/cm for 20 msec each by an Electro Cell Manipulator 2001 (BTX). Reconstructed embryos without visible somatic cells were determined as fused eggs 1 hr after the fusion pulse. For activation, 4 hr after electrofusion, the fused eggs were activated with 5 μM ionomycin for 5 min, followed by treatment with 2.5 mM 6-dimethyl-aminopurine in CR1aa supplemented with 10% FBS for 3.5 hr at 38.5° in 5% CO_2_ in air. Thereafter, the embryos were cultured as were the IVF embryos described previously. Blastocysts were obtained 6–8 d post-NT. We counted the number of nuclei in individual blastocysts after live Hoechst staining and selected only healthy-looking blastocysts with 60–80 blastomeres. For donor cell preparation, ear skin fibroblasts were obtained from an adult female and maintained three passages in the culture condition as described previously (Koo *et al.* 2002). Cumulus cells were freshly removed from cumulus−oocyte complexes and used in nuclear transfer the same day.

### Isolation and amplification of whole transcripts

Messenger RNAs were isolated from single bovine blastocysts with the use of Dynabeads mRNA DIRECT Kit (Invitrogen). The purified mRNAs were used as templates in random cDNA synthesis using SuperScript III reverse transcriptase (Invitrogen). For this, we used a unique adaptor, 5′- GTGGTGTGTTGGGTGTGTTTGAGTCNNNN-3′, that contained 3′ random quadruple sequence. To this adaptor, we inserted the Mly1 restriction-enzyme recognition sequence (5′-GAGTC(N)_5_-3′). With this MlyI-anchored adaptor, first-strand cDNA synthesis was performed using a sequential program as follows: 18° for 10 min, 25° for 10 min, 37° for 30 min, 42° for 10 min, and 70° for 20 min. Then, T4 DNA polymerase (NEB) was added to the cDNA synthesis mixture and further incubated at 37° for 1 hr. For amplification of the cDNA library, PCR was performed with 20 cycles of 94° for 2 min, 70° for 5 min, using a primer, 5′-GTGGTGTGTTGGGTGTGTTT-3′. The amplified PCR products were digested by *Mly*I enzyme (NEB) overnight and then purified using Qiaquick PCR Purification Kit (QIAGEN).

### Library construction for RNA-seq

A sequencing library for each single blastocyst transcriptome was constructed using TruSeq DNA Sample Prep Kit (Illumina) according to the manufacturer’s instructions with some minor modifications. A 200-ng amplicon in 25 μL of volume was incubated with 20 μL of End-repair Mix (Illumina) and 5 μL of Resuspension Buffer (RB, Illumina) for 2 hr at 30°. The reaction mixtures were purified with AMPure XP Beads (Beckman Coulter) according to manufacturer’s instructions and eluted with 15 μL of RB. 3′-adenylation was performed on the purified DNAs by incubating with 12.5 μL of A-tailing Mix (Illumina) and 2.5 μL of RB for 2 hr at 30°. Adapters with unique indexes were ligated onto the 3′-adenylated DNAs by adding 1 μL of DNA Adapter Index (Illumina), 2.5 μL of DNA Ligase Mix (Illumina), and 4 μL of RB and incubating overnight at 16°. The following day, ligation mixtures were purified twice with beads and eluted with 30 μL of RB. For size selection, the adapter ligated amplicons were loaded on 2% Agarose Dye Free Gel Cassettes (Sage Science) and amplicons within size of 200−500 bp were isolated with Blue Pippin DNA size selection system (Sage Science). Finally, the size-selected amplicons were enriched via PCR. Then, 5 μL of each amplicons was mixed with 25 μL of PCR Master Mix (Illumina), 5 μL of PCR Primer Cocktail (Illumina), and 20 μL of RB. Fifteen cycles of PCR was performed in C1000 Touch Thermal Cycler (BioRad) with a following program: predenaturation at 98° for 30 sec, 10 cycles of 98° for 10 sec, 60° for 30 sec, 72° for 30 sec, final extension at 72° for 5 min, and hold at 4°. The PCR products were purified with Qiaquick PCR Purification Kit (QIAGEN) according to the manufacturer’s instructions. The libraries were loaded on 2% agarose gels to validate their sizes and quantities. For more accurate quantification of libraries, quantitative polymerase chain reaction (qPCR) was performed. Then, 2 μL of each library was mixed with 10 μL of Topreal qPCR 2x PreMix (Enzynomcis), 10 μM each qPCR Primer 1.1 and 1.2, and 8 μL of DW in a MicroAmp Optical 8-strip tube (Applied Biosystems). Additionally, control libraries of known quantities were prepared in various concentrations for standard curve generation. All the samples and controls were performed in triplicate, and qPCR was performed in 7500 Real-Time PCR System (Applied Biosystems). A standard curve was generated with Ct values of control libraries, and by use of the equation of the curve, the concentration of each sample library was calculated. Then, the same amount of each library was pooled in a 1.5-mL tube before sequencing.

### Gene expression estimation and differential expression analysis

Raw read sequences from single blastocysts were mapped on the reference genome (UMD3.1) with TopHat, and then the number of mapped reads was counted with the ‘HTSeq’ python package with the ‘intersection-strict’ option. Two different approaches, DESeq and edgeR, were used to comprehensively estimate gene expression and identify differentially expressed genes (DEGs). Raw count data from HTSeq were merged into a single data table and processed with DESeq and edgeR. Normalized gene expression profiles of single blastocysts were estimated and consequently used in differential expression analysis.

### Quantitative real-time PCR

For quantitative real-time PCR validation of DEGs, cDNA libraries were prepared from each of six male IVF or fSCNT blastocysts and pooled before use in PCR. To obtain male fSCNT blastocyst, male ear skin fibroblasts were used as donor cells. To select male IVF blastocysts, genomic DNAs of individual blastocysts saved in the course of total RNA isolation were used for gender identification by PCR sexing (Rattanasuk *et al.* 2011) and only those that were identified as males were used for real-time PCR analysis. One microliter (one-twentieth volume) of IVF- or SCNT-pooled cDNAs were mixed with 10 μL of TOPreal qPCR 2X PreMIX (Enzynomics), 10 μM DEGs specific primers, and 8 μL of DW. qPCR was performed in a 7500 Real-Time PCR System (Applied Biosystems). All the experiments were duplicated. The list of PCR primers is as follows: ATCAACTGACCCTGGCTGAC and GAGATAGCGCCAGACTCCTG for the *CLIC2* gene; TCATGACGGTCAACGAGTTC and GATGCTGATGTCCTGCTTCA for *JMJD5*; TCCACCTGGTCCCTTTGTAG and TGGAGTGAAACCAAGGGAAG for *PRAME*; TGTCCCCAAGACAAGAGACC and CGAATGCCAGAGGAAAAAGA for *CCR7*; CGGAGAGCCTGACTTACTGG; and TGTGATCTGCAAGGCACATT for *DOK5*.

### Generation of a Circos plot for differential expression profile

For Circos representation of differential expression (Krzywinski *et al.* 2009), significant DEGs (*P*-value < 0.05) from DESeq in cSCNT or fSCNT against IVF were identified, and by use of their fold-change values and genomic location data, the differential expression profile was visualized. DEG-dense regions (five or more DEGs per 1-Mb window) were determined by sliding window analysis (2-Mb window, 1-Mb step) of DEG counts throughout the somatic chromosomes in cSCNT or fSCNT *vs.* IVF and presented on the Circos plot.

### Data availability

File S1 contains normalized global expression profile data of all blastocysts. File S2 contains single blastocyst expression profiles of pluripotency genes (PG), trophectoderm genes (TEG), developmental regulator genes (DRG), and epigenetic modifier genes (EMG).

## Results

**Figure 1:**
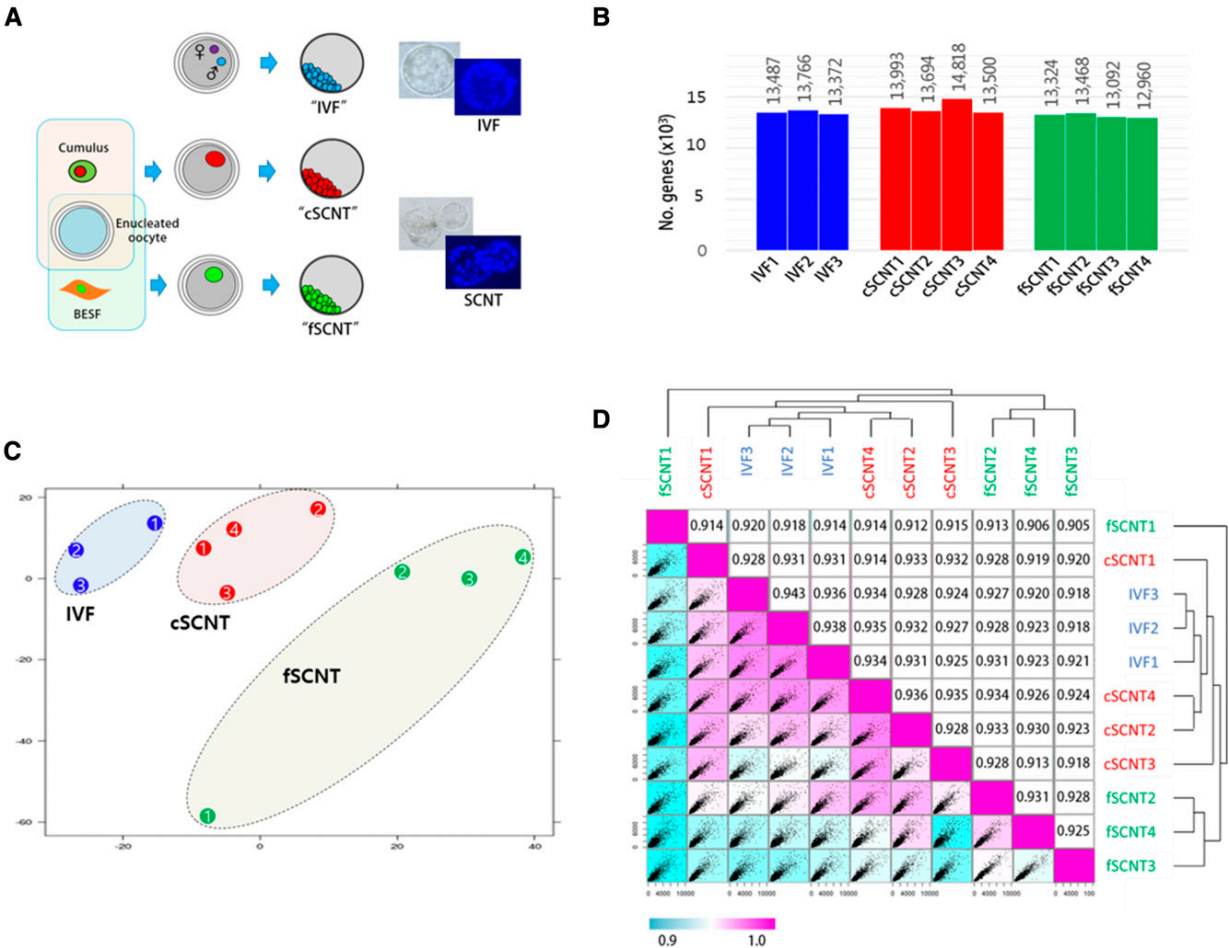
Overview of cloned blastocyst production and their expression profiles. (A) Production of blastocysts derived by *in vitro* fertilization (IVF) or somatic cell nuclear transfer (SCNT). IVF embryos were obtained by fertilization of oocytes from unrelated cows with semen from a single bull. Cumulus cells (female) and adult ear skin fibroblasts (BESF, female) were donor cells used to obtain “cSCNT” and “fSCNT” blastocysts, respectively. IVF and SCNT embryos were cultured under identical conditions. Blastocysts were live-stained with Hoechst to count the number of blastomeres. (B) Number of detected genes expressed in each blastocyst. (C) Principal component analysis. Individual blastocysts that belong to IVF (blue), cSCNT (red), and fSCNT (green) groups are numbered. (D) Unsupervised Spearman correlation analysis. Expressional correlation coefficients (r^2^) between single blastocysts were calculated and plotted as a scatter matrix. Diagonally arranged pink boxes indicate the highest correlation (r^2^ = 1), and correlation decreases as the box color turns blue.

We investigated whole transcript profiles in bovine blastocysts, which we derived from IVF and SCNT. In SCNT, reprogramming markedly differs between different types of donor cells (Beyhan *et al.* 2007; Fukuda *et al.* 2010); thus, donor cell type is important for successful cloning (Tian *et al.* 2003). We used two different adult cells: cumulus cells and ear skin fibroblasts, from which cSCNT and fSCNT blastocysts were derived, respectively. To reduce variation among samples, morphologically healthy, mid-expanded blastocysts harboring only 60–80 cells were chosen for analysis after live Hoechst staining (Figure 1A).

To obtain cDNAs from single blastocysts, we used a PCR-based random amplification of whole transcripts from a minute amount of RNA (Gonzalez-Roca *et al.* 2010) and, with these amplicons, RNA-seq libraries were constructed. The resulting libraries were sequenced with HiSeq-2000 and 12.7 million paired-end reads, on average, were generated. Using a splice junction mapper called “TopHat” (Trapnell *et al.* 2012), we mapped raw reads onto a reference genome, *Bos taurus* UMD3.1.72 (UMD3.1) from ENSEMBL, which possesses 26,740 annotated genes in total. Approximately 63% (8 million reads) of the raw reads from each blastocyst were mapped to putative exons or their flanking regions. In total, 16,381 (81.9%) of 19,994 coding genes were detected. The number of genes expressed in each blastocyst was 13,372–13,766 for the IVF group, 13,500–14,818 for cSCNT, and 12,960–13,468 for fSCNT (Figure 1B; see also File S1). There was no significant difference in the number of expressed genes between IVF and either cSCNT or fSCNT at *P*-value <0.05 level.

With the transcriptome data, correlation analysis was performed. Principal component analysis showed that the blastocysts were, overall, grouped according to their origins and, cSCNT blastocysts were closer to IVF blastocysts than fSCNT blastocysts (Figure 1C). The Spearman correlation analysis produced a similar result (Figure 1D). As represented by color, the cSCNT group was closer to the IVF group than the fSCNT group, and cSCNT3 and fSCNT2 were peculiar and deviated the most from other cSCNT and fSCNT blastocysts, respectively. Coefficient values within each blastocyst group showed that, compared with IVF blastocysts (*r^2^* = 0.936−0.943), cSCNT (0.928−0.936), and fSCNT (0.905−0.931) were less correlated with each other. Among SCNT blastocysts, fSCNT1 (0.914−920) was the least correlated with the three IVF blastocysts, whereas cSCNT4 (0.934−0.935) was, conversely, the most.

**Figure 2:**
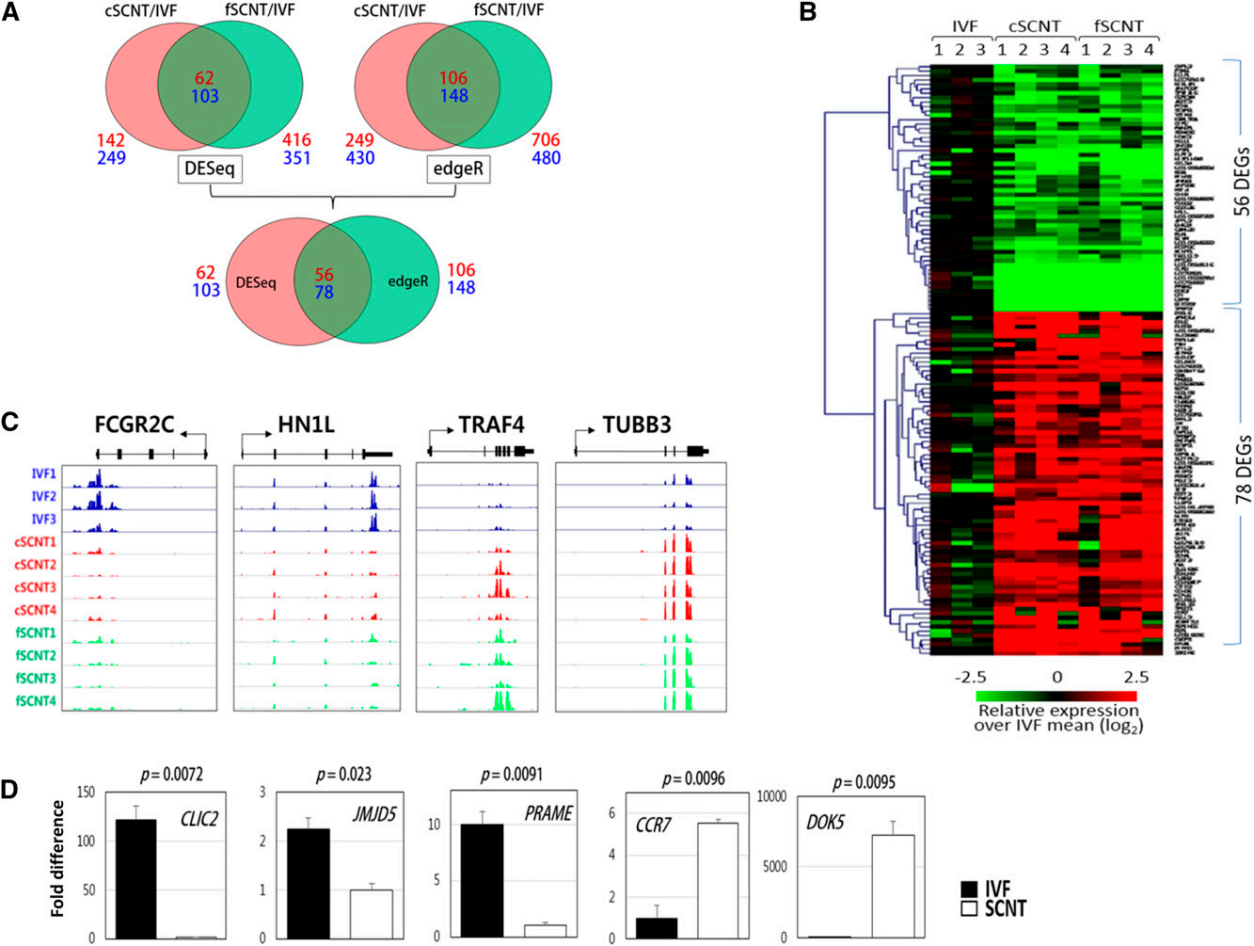
Identification of differentially expressed genes (DEGs) among blastocyst groups. (A) Identification of DEGs. DESeq found 62 underrepresented (SCNT-low) and 103 overrepresented (SCNT-high) DEGs in c- and fSCNT-high DEGs, respectively, whereas edgeR detected 106 SCNT-low and 148 SCNT-high DEGs (top). Finally, the 56 SCNT-low and 78 SCNT-high DEGs that were consistent between DESeq and edgeR are identified (bottom). Numbers in red and blue designate the number of SCNT-low and -high DEGs, respectively. IVF, *in vitro* fertilization. SCNT, somatic cell nuclear transfer. (B) Heatmap of DEGs between IVF and SCNT blastocysts showing relative expressions over the mean of IVF single blastocysts. (C) Representative coverage plots. Read coverage of some SCNT-low (*FCGR2C* and *HN1L*) and SCNT-high (*TRAF4* and *TUBB3*) DEGs are shown using an Integrated Genome Viewer (IGV). (D) Quantitative real-time polymerase chain reaction (PCR) validation. Differential expression of SCNT-low (*CLIC2*, *JMJD5*, and *PRAME*) and SCNT-high (*CCR7* and *DOK5*) genes were validated by real-time PCR by the use of cDNAs from male IVF (solid bar) or fSCNT (blank bar) blastocysts. Error bars, standard deviation. *P*-values (*t*-test) are denoted.

To identify DEGs between the blastocyst groups, we used two different analysis methods: DESeq and edgeR. DESeq found 62 and 103 candidate genes that were underrepresented and overrepresented, respectively, in both SCNT groups (fold change of >2 and *P* < 0.05; Figure 2A). In the same way, edgeR detected 106 underrepresented and 148 overrepresented candidate genes. To obtain highly reliable DEGs against the false-positive, only genes that were commonly detected by both DESeq and edgeR were selected. Thus, we finally identified 134 DEGs in total, including 56 underrepresented (SCNT-low) and 78 overrepresented (SCNT-high) in both SCNT groups. Figure 2B shows the heatmap of the 134 DEGs among the blastocysts of different groups. The identified SCNT-low and SCNT-high DEGs are listed in Table 1 and Table 2, respectively. Representative read coverage plots are shown in Figure 2C. By quantitative real-time PCR analysis, we were convinced that the identified DEGs were differentially expressed between IVF and fSCNT blastocysts that were freshly produced for validation (Figure 2D). Meanwhile, in the cSCNT and fSCNT group comparisons, 586 DEGs were detected by DESeq, which reflects different biological performances between donor cells (Supporting Information, Figure S1, File S1, and File S2). Gene Ontology (David Functional Annotation Tool) classification was used for categorizing the identified DEGs. No functional categories were enriched in SCNT-low DEGs. Among the SCNT-high DEGs, regulation of apoptosis (*AIFM2*, *HSPA1A*, *DDIT3*, *NR4A1*, *TRAF4*) and negative regulation of caspase activity (*HSPA1A*, *NR4A1*) under the Biological Process heading were overrepresented (*P* < 0.05).

**Table 1:** Down-regulated DEGs in cSCNT and fSCNT blastocysts against IVF

Symbol	refSeq	Chr	cSCNT/IVF		fSCNT/IVF	
			Fold Change	*P*-Value	Fold Change	*P*-Value
*NPSR1*	NM_001192977	4	−9.85	8.3E-04	−10.27	5.2E-04
*LOC789301*	XM_003587135	17	−9.07	2.2E-06	−9.07	9.6E-07
*LOC100336994*	XR_083901	17	−8.84	5.9E-05	−11.09	8.2E-05
*UTY*	XM_002700446	X	−8.16	4.1E-22	−8.16	2.3E-23
*DDX3Y*	NM_001172595	X	−7.27	3.4E-17	−10.30	4.1E-17
*GLRB*	NM_174071	17	−6.88	5.6E-04	−5.06	9.7E-05
*USP9Y*	NM_001145509	X	−6.40	4.2E-10	−6.40	1.2E-10
*EIF2S3Y*	XM_002700462	X	−5.47	9.0E-06	−5.47	4.5E-06
*PRAME*	NM_001077979	17	−4.35	5.5E-03	−4.35	3.9E-03
*LOC100847116*	XR_139203	1	−4.35	7.6E-03	−5.22	3.6E-03
*LOC784883*	XM_002683623	5	−4.16	1.1E-02	−4.16	7.9E-03
*VSTM4*	XM_002698947	28	−3.95	5.9E-03	−4.17	3.1E-03
*ZRSR2Y*	XM_003588207	X	−3.91	2.2E-02	−3.91	1.7E-02
*LOC789418*	XM_002707755	18	−3.63	1.4E-02	−3.75	6.0E-03
*LOC100848896*	XM_003587423	19	−3.23	4.2E-03	−2.60	1.9E-02
*ECE1*	NM_181009	2	−3.04	1.2E-02	−2.38	2.3E-02
*CCL24*	NM_001046596	25	−2.95	2.4E-02	−3.33	5.2E-03
*FCGR2C*	NM_001109806	3	−2.89	3.7E-05	−3.01	3.5E-05
*SEMA3E*	XM_003585955	4	−2.83	6.8E-03	−2.57	1.5E-02
*LOC100848332*	XM_003587324	18	−2.32	2.0E-04	−1.54	9.2E-03
*ABCC2*	XM_002698487	26	−2.21	2.1E-02	−1.93	2.2E-02
*HIST1H2AJ*	XM_002697473	23	−2.07	1.7E-03	−4.54	3.1E-08
*FAM190A*	NM_001076437	6	−2.05	3.8E-02	−2.08	2.1E-02
*RPS20*	NM_001034438	14	−1.86	3.2E-04	−1.98	1.2E-04
*SPATA16*	XM_002684936	1	−1.86	6.5E-03	−2.54	1.6E-04
*CERCAM*	NM_001102035	11	−1.84	8.9E-03	−1.47	4.2E-02
*LOC100337329*	XM_003586293	7	−1.82	1.3E-02	−1.87	1.3E-02
*TBC1D19*	NM_001076938	6	−1.79	3.0E-03	−1.71	4.0E-03
*LOC100848324*	XR_139547	17	−1.78	1.2E-02	−2.49	4.3E-04
*FLRT3*	NM_001192674	13	−1.66	1.0E-02	−2.14	1.3E-03
*PDCD2*	NM_001046109	9	−1.60	4.8E-03	−1.07	4.1E-02
*BIVM*	NM_001206453	12	−1.59	2.0E-02	−1.38	2.7E-02
*MST4*	NM_001163786	X	−1.55	1.3E-03	−1.69	3.4E-05
*IL1R1*	NM_001206735	11	−1.54	9.3E-03	−1.22	3.0E-02
*ANXA3*	NM_001035325	6	−1.49	2.2E-02	−1.44	1.3E-02
*HDAC3*	NM_001206243	7	−1.46	9.1E-03	−1.20	3.6E-02
*CMPK2*	XM_002691489	11	−1.43	4.6E-04	−1.01	1.4E-02
*DHX29*	NM_001206134	20	−1.41	1.0E-04	−1.22	1.6E-03
*SCARB1*	NM_174597	17	−1.40	6.0E-03	−1.23	7.8E-03
*ZNF22*	NM_001077108	28	−1.39	3.3E-02	−1.67	1.3E-02
*PCNA*	NM_001034494	13	−1.29	9.2E-03	−1.27	7.5E-03
*MINPP1*	NM_001038575	26	−1.28	2.8E-03	−1.08	1.2E-02
*CCDC25*	NM_001035044	8	−1.25	3.4E-02	−1.35	4.2E-02
*NOL11*	NM_001034525	19	−1.25	5.7E-04	−1.02	5.0E-03
*MTHFS*	NM_001075616	21	−1.23	1.8E-02	−1.66	6.0E-04
*HN1L*	NM_001081546	25	−1.21	7.8E-04	−1.45	1.1E-04
*RANBP1*	NM_001034586	17	−1.20	7.0E-03	−1.06	2.3E-02
*CAMK2D*	NM_001046333	6	−1.16	2.6E-03	−1.15	2.1E-03
*MLH1*	NM_001075994	22	−1.11	2.4E-02	−1.29	5.7E-03
*PSMB1*	NM_001038539	9	−1.07	2.7E-02	−1.08	3.6E-02
*HIF1AN*	NM_001083443	26	−1.07	1.7E-02	−1.40	2.9E-03
*ANP32E*	NM_001075306	3	−1.07	1.3E-02	−1.09	4.5E-03
*ANKRD27*	NM_001102532	18	−1.04	1.2E-03	−1.47	1.4E-06
*APPL2*	NM_001046206	5	−1.02	5.6E-03	−1.05	3.4E-03
*CHKB*	NM_001206094	5	−1.01	4.0E-02	−1.26	1.1E-02
*OIP5*	NM_001205692	10	−1.00	1.9E-02	−1.38	4.5E-03

DEGs, differentially expressed genes; cSCNT, cumulus cells somatic cell nuclear transfer; fSCNT, adult ear skin fibroblasts somatic cell nuclear transfer; IVF, *in vitro* fertilization.

**Table 2:** Up-regulated DEGs in cSCNT and fSCNT blastocysts against IVF

Symbol	refSeq	Chr	cSCNT/IVF		fSCNT/IVF	
			Fold Change	*P*-Value	Fold Change	*P*-Value
*CCR7*	NM_001024930	19	7.47	2.5E-02	6.92	4.0E-02
*LOC618696*	XR_083836	13	6.64	9.8E-03	5.00	4.1E-02
*CADPS*	NM_001076020	22	5.46	3.2E-05	4.58	8.9E-04
*PTPRD*	XM_003586379	8	5.18	3.0E-03	5.25	3.7E-03
*ARMCX4*	XM_003588124	X	5.09	3.3E-04	2.97	4.3E-02
*LOC526966*	XM_002700268	X	5.03	1.9E-02	5.98	4.9E-03
*SPECC1*	XM_002695819	19	5.02	1.8E-02	5.09	1.8E-02
*LOC100847564*	XR_139737	X	4.78	4.8E-02	5.81	2.1E-02
*SLC38A5*	NM_001015580	X	4.78	8.8E-03	4.38	2.1E-02
*FGF16*	NM_001192777	X	4.77	5.6E-03	2.88	4.6E-02
*LOC100140788*	XR_084090	27	4.64	1.2E-03	3.11	1.2E-02
*LOC781710*	XM_002686247	3	4.56	3.4E-04	3.56	4.3E-03
*DOK5*	NM_001075939	13	4.55	6.4E-03	5.46	2.0E-03
*SOHLH2*	XM_002691802	12	4.55	1.7E-05	4.01	9.2E-05
*ULBP3*	NM_001103233	17	4.29	1.7E-02	3.91	2.4E-02
*LOC782781*	NM_001168605	3	4.26	3.8E-03	3.87	7.3E-03
*NELL2*	NM_001102084	5	4.25	1.6E-02	3.59	4.0E-02
*FER1L6*	XM_002692627	14	4.19	1.8E-02	5.38	2.1E-03
*ADAMTS4*	NM_181667	3	3.76	2.9E-02	4.23	2.1E-02
*PPFIA2*	XM_002687191	5	3.74	1.4E-02	3.09	2.9E-02
*LOC787130*	XM_002700111	X	3.64	8.8E-05	3.49	2.1E-04
*NR4A1*	NM_001075911	5	3.38	7.8E-04	2.03	4.9E-02
*PAH*	NM_001046058	5	3.31	2.4E-02	4.05	8.1E-03
*EGR3*	XM_003586409	8	3.20	1.6E-02	4.06	7.7E-03
*ISCA2*	NM_001038683	10	3.11	1.7E-02	3.49	4.2E-03
*ALDOC*	NM_001097984	19	3.02	6.3E-03	2.37	2.9E-02
*LOC525414*	XM_003587677	23	2.99	5.8E-03	3.26	2.7E-03
*TUBA3E*	NM_001038163	17	2.98	3.7E-04	3.16	2.3E-04
*SAT1*	NM_001034333	X	2.87	8.1E-03	2.22	4.5E-02
*SYTL2*	NM_001102278	29	2.87	2.2E-02	6.02	2.3E-07
*TEK*	NM_173964	8	2.75	1.3E-03	2.72	1.1E-03
*COL6A2*	NM_001075126	1	2.70	3.8E-02	2.79	3.4E-02
*SLC16A6*	NM_001192672	19	2.68	6.6E-06	2.41	5.2E-05
*C8H9orf64*	NM_001076978	8	2.53	2.4E-02	2.90	8.8E-03
*FBP1*	NM_001034447	8	2.44	3.7E-02	2.64	3.4E-02
*LOC100335242*	XM_003585627	1	2.42	4.2E-04	1.60	1.7E-02
*SYCP2*	XM_002692264	13	2.41	3.0E-03	2.03	1.7E-02
*H1F0*	NM_001076487	5	2.40	2.8E-02	2.51	2.0E-02
*HSPA1A*	NM_203322	23	2.40	1.3E-03	3.29	7.4E-05
*ZNF133*	NM_001110090	13	2.38	2.2E-02	2.23	2.5E-02
*IMPDH1*	NM_001077841	4	2.16	9.5E-04	1.91	4.0E-03
*HAVCR1*	XM_002683662	7	2.09	1.1E-03	1.90	3.6E-03
*AKIP1*	NM_001034203	15	1.92	3.8E-02	2.13	2.3E-02
*FHOD3*	NM_001191215	24	1.92	2.2E-02	2.14	1.4E-02
*AIFM2*	NM_001040556	28	1.84	1.9E-02	1.75	3.9E-02
*TUBB3*	NM_001077127	18	1.78	7.4E-07	1.48	1.2E-05
*LOC100848375*	XR_139401	11	1.78	2.2E-03	1.74	4.3E-03
*XIST*	NR_001464	X	1.76	8.5E-04	2.23	4.2E-05
*ZSWIM6*	XM_002696311	20	1.74	4.3E-05	1.26	5.4E-03
*FMNL3*	NM_001191506	5	1.72	2.0E-02	1.88	1.2E-02
*CHN1*	NM_001075349	2	1.72	1.6E-02	1.96	8.9E-03
*CDH26*	XM_002692262	13	1.63	1.8E-06	1.59	2.9E-06
*CDKN2AIP*	XM_003587947	27	1.59	4.0E-04	1.09	1.1E-02
*HAUS7*	NM_001102277	X	1.57	3.3E-03	1.99	5.8E-04
*DDIT3*	NM_001078163	5	1.54	6.2E-03	1.52	3.0E-03
*SERINC5*	XM_002690460	10	1.45	2.2E-02	2.23	1.8E-03
*CSTF2*	NM_174685	X	1.41	3.3E-04	1.09	2.2E-03
*JMY*	XM_002690458	10	1.30	4.0E-02	1.66	6.2E-03
*GBA*	NM_001046421	3	1.23	1.7E-04	1.32	8.4E-05
*GRIPAP1*	NM_001193106	X	1.22	2.1E-03	1.29	3.3E-03
*ADCY2*	XM_002696432	20	1.20	1.8E-02	1.35	5.7E-03
*RBMX2*	NM_001206548	X	1.19	8.2E-04	1.39	9.8E-05
*SLITRK2*	XM_002699621	X	1.19	2.0E-03	1.38	1.0E-03
*CHCHD7*	NM_001130759	14	1.18	8.6E-03	1.41	4.9E-03
*PM20D1*	NM_001038100	16	1.17	1.1E-02	1.22	1.4E-02
*NDUFB11*	NM_001033620	X	1.15	2.6E-03	1.43	9.1E-05
*CNNM4*	NM_001205896	11	1.15	7.6E-03	1.23	5.2E-03
*BCAP31*	NM_001014941	X	1.15	6.2E-05	1.13	2.3E-04
*HECW2*	XM_002685474	2	1.14	1.3E-02	1.19	8.4E-03
*ATG3*	NM_001075364	1	1.14	1.9E-02	1.22	9.5E-03
*TRAF4*	NM_001101280	19	1.11	1.7E-02	1.23	8.1E-03
*PELI2*	XM_002690952	10	1.11	7.4E-03	1.61	3.7E-04
*WDR45*	NM_001076151	X	1.09	2.1E-04	1.06	2.2E-04
*COQ10B*	NM_001075654	2	1.07	1.8E-02	1.39	3.8E-03
*SRGAP2*	NM_001205865	16	1.05	1.7E-02	1.30	4.9E-03
*ENPP1*	NM_001206212	9	1.05	2.2E-02	1.21	4.6E-03
*SCNM1*	NM_001034254	3	1.04	9.1E-03	1.14	3.0E-03
*LOC782021*	XR_084028	22	1.01	3.6E-03	1.78	5.4E-07

DEGs, differentially expressed genes; cSCNT, cumulus cells somatic cell nuclear transfer; fSCNT, adult ear skin fibroblasts somatic cell nuclear transfer; IVF, *in vitro* fertilization.

**Figure 3:**
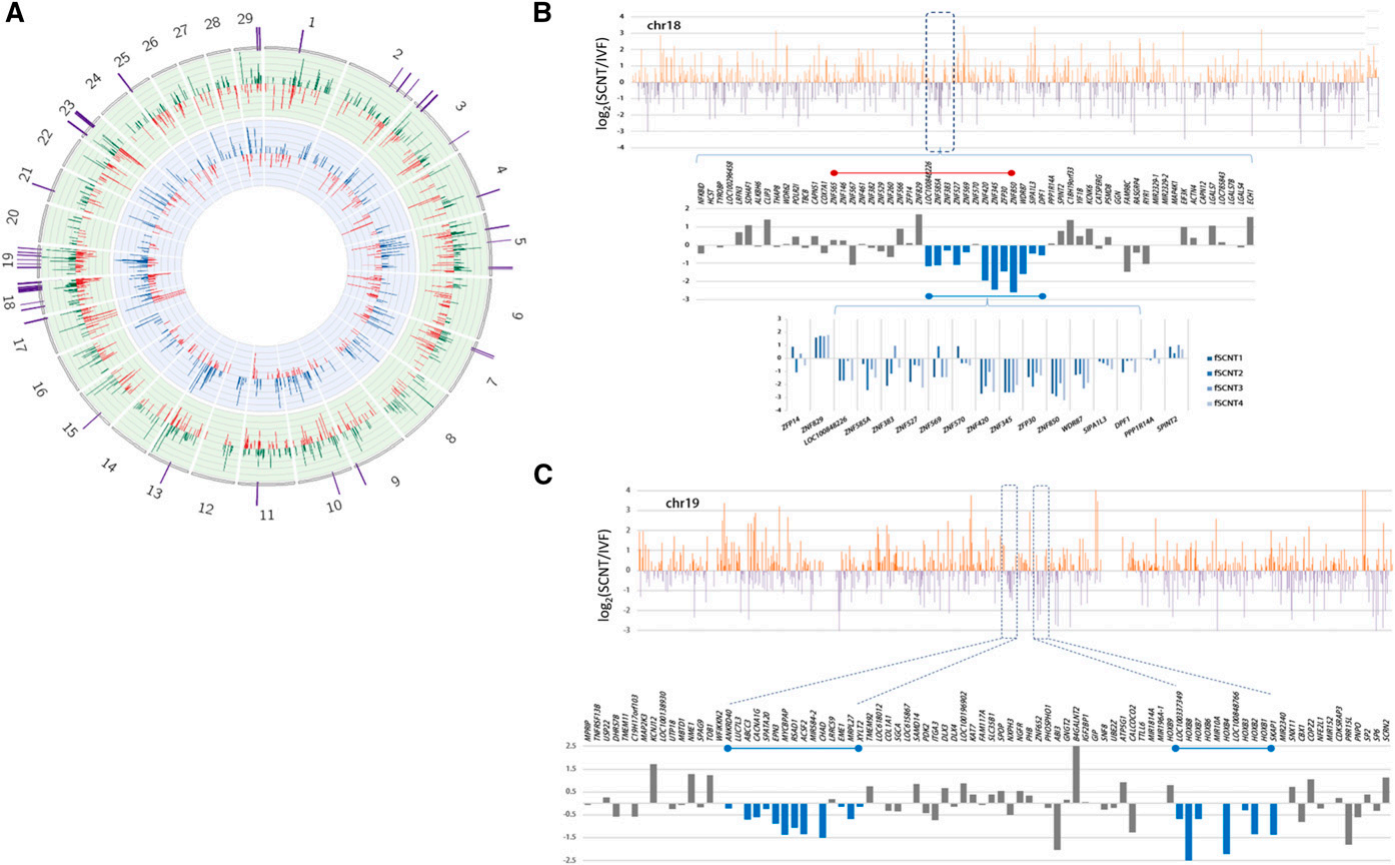
Representatives of coordinately regulated genomic regions in somatic cell nuclear transfer (SCNT) blastocysts. (A) Circos plot for differential expression profile (DEP). The inner circle on blue background represents DEP between cSCNT (blue bars) and *in vitro* fertilization (IVF; red) whereas the green circular layer indicates DEP between fSCNT (green bars) and IVF (red). Fold changes are between −5 and 5, and only significant differentially expressed genes (DEGs) (*P*-value < 0.05) are included in the plot. DEG-dense regions are depicted as purple bars on the plot surface. (B−C) Plots of DEGs in fSCNT blastocysts in chromosomes 18 (B) and 19 (C). All genes in chr18 are sorted in the order of the genomic location, and their fold-change values [log_2_(fSCNT/IVF)] were shown in the bar graph. Upper orange and lower purple bars indicate up- and down-regulation, respectively. Some representative genomic regions that seem to be under coordinate regulation are boxed and enlarged below and, in these regions, coordinately regulated genes are indicated in blue.

To search for poorly reprogrammed genomic regions in SCNT blastocysts, a sliding window analysis using genomic location of DEGs was performed. As shown on the Circos plot (Krzywinski *et al.* 2009) in Figure 3A, we found DEG peaks prominently enriched in chromosomes 18 and 19, and we inspected these chromosomes further for genomic loci where adjacent genes were under a coordinate regulation in SCNT blastocysts and expressed in a different fashion from those of IVF genes (Figure 3B). The in-depth screening led to the identification of a genomic region (∼47 Mb) in chromosome 18 where a dozen adjacent genes were coordinately down-regulated (Figure 3C). Interestingly, this 400-kb genomic region includes a part of zinc-finger protein (ZNF) gene cluster in which, of 19 ZNF genes in the cluster, nine adjacent ZNF genes were down-regulated in the fSCNT group; this down-regulation was very consistent in all fSCNT blastocysts examined. In cSCNT blastocysts, however, the effect was not that much clear as in fSCNT and looked inconstant among individual cSCNT blastocysts (Figure S2). We also found genomic stretches belonging to coordinate regulation in chromosome 19 as representatively shown in Figure 3D. HOXB family genes are clustered 200 kb span in chromosome 19 (38.5 Mb) and several of them such as *HOXB2*, *HOXB4*, *HOXB7*, and *HOXB8* were markedly down-regulated in fSCNT blastocysts. It would be interesting to study the functional significance of HOXB gene expression at the blastocyst stage, and the consequence of substantial underexpression of HOXB genes at this stage or later in fSCNT embryo development.

**Figure 4:**
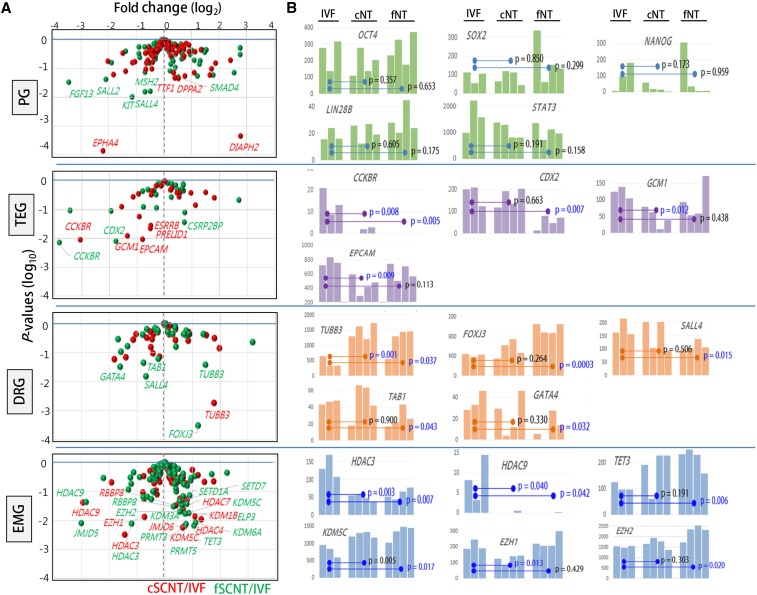
Differential expressions of developmentally important genes between *in vitro* fertilization (IVF) and somatic cell nuclear transfer (SCNT) blastocysts. (A) Volcano plots of relative expression of each category of genes in cSCNT (red) and fSCNT (green) over IVF mean. Differentially expressed genes (DEGs) with *P*-values of <0.05 are labeled with gene symbols. PG, 100 pluripotency-related genes; TEG, 27 trophectoderm development-involved genes; DRGs, 51 developmental regulators; EMGs, 89 epigenetic modifiers. (B) DEGs between IVF and either cSCNT or fSCNT blastocysts. Statistical differences between the blastocyst groups are indicated by *p*-values (*t*-test).

The blastocyst is a key developmental stage featured by the gaining of pluripotency and the beginning of lineage differentiation into inner cell mass and trophectoderm cells. We probed into the transcriptome data, focusing on four categories of genes that are intimately related to blastocyst development (Figure 4A; see also File S2): 100 pluripotency-related genes (Deb *et al.* 2008; Zhao *et al.* 2012), 27 trophectoderm development−involved genes [TEGs; (Bai *et al.* 2012)], genes encoding 51 developmental regulators (DRGs; www.sabiosciences.com/rt_pcr_product/HTML/PAMM-508A.html]), and genes encoding 89 epigenetic modifiers (EMGs), which we selected from extensive literature study. Of the 267 genes examined, 19 (7.0%) and 27 (10.0%) were differentially expressed between IVF and either cSCNT or fSCNT, respectively, at *P* < 0.05 (Table 3). PGs were, overall, similarly expressed among the blastocyst groups; expression levels of well-known PGs, including *OCT4*, *SOX2*, *LIN28B*, *STAT3*, and *NANOG*, are represented in Figure 4B. This result is consistent with results of a previous study that used microarray data in mouse cloned blastocysts (Fukuda *et al.* 2010), although it differs from other studies where pluripotency genes, including *Oct4*, were misregulated in cloned embryos (Boiani *et al.* 2002; Bortvin *et al.* 2003). Of particular interest were variable expression levels of *NANOG* among blastocysts, although the implication of it is unknown at present.

**Table 3:** Differentially expressed genes in different categories important for blastocyst development

Category (No. Genes)	cSCNT/IVF		fSCNT/IVF	
	cSCNT-Low	cSCNT-High	fSCNT-Low	fSCNT-High
PGs (100)	*EPHA4*	*DIPH2*, *DPPA2*, *TTF1*	*FGF13*, *SALL2*, *KIT*, *SALL4*, *MSH2*	*SMAD4*
TGs (27)	*CCKBR*, *GCM1*, *PCAM*, *PRELID1*, *ESRRB*	*−*	*CCKBR*, *CDX2*	*CSRP2BP*
DRGs (51)	*−*	*TUBB3*	*GATA4*, TAB1	*FOXJ3*, *TUBB3*
EMGs (89)	*HDAC9*, *HDAC3*, *EZH1*	*HDAC4*, *KDM1B*, *JMJD6*, *HDAC7*, *KDM5C*, *RBBP8*	*JMJD5*, *HDAC9*, *HDAC3*	*KDM6A*, *TET3*, *ELP3*, *SETD1A*, *PRMT5*, *PRMT3*, *KDM5C*, *KDM3A*, *EZH2*, *SETD7*, *RBBP8*

*P*-value < 0.05. cSCNT, cumulus cells somatic cell nuclear transfer; fSCNT, adult ear skin fibroblasts somatic cell nuclear transfer; IVF, *in vitro* fertilization; PGs, pluripotency-related genes; TGs, genes involved in TE development; DRGs, genes encoding developmental regulators; EMGs, genes encoding epigenetic modifiers.

Meanwhile, a large number of TEGs were differentially expressed in SCNT embryos: five (*CCKBR*, *GCM1*, *EPCAM*, *PRELID1*, and *ESRRB*) and three (*CCKBR*, *CDX2*, and *CSRP2BP*) in the cSCNT and fSCNT groups, respectively (Table 3). These data suggest a potential defect in extraembryonic tissue development in SCNT blastocysts and support the notion that poor placental development is one of the major problems associated with SCNT embryo mortality and a consistent feature of SCNT pregnancies (Constant *et al.* 2006; De Sousa *et al.* 2001; Fletcher *et al.* 2007; Hill *et al.* 2000; Kang *et al.* 2002; Oda *et al.* 2010; Palmieri *et al.* 2008). Notably, all differentially expressed TEGs, except for *CSRP2BP*, were underrepresented in SCNT blastocysts. We also noticed that aberrantly expressed TEGs were not centered onto particular SCNT blastocysts but were instead spread among the SCNT blastocysts. If the aberrancy in TEG expression is attributed to the stochastic event of reprogramming, then the first question answered should be, “why are TEGs especially prone to such stochastic reprogramming?”

No noticeable difference was detected in expression of 51 DRGs between the blastocyst groups. As observed in the PGs, there were slightly more differentially expressed DRGs in the IVF-fSCNT set; *FOXJ3*, *TAB1*, *GATA4*, and *TUBB3* were abnormally expressed in fSCNT blastocysts, and *TUBB3* appeared in both cSCNT and fSCNT groups. Because SCNT embryo requires a proper expression of imprinting genes for normal development (Okae *et al.* 2014; Suzuki *et al.* 2011), we inquired into imprinting genes. Mean expression levels of 28 cow imprinting genes were not very different between the groups, although we noted variable levels of expression in some genes (*MEST*, *NNAT*, *PEG3*, *PEG10*, *SNRPN*, etc.) among blastocysts in each group (Figure S3). It remains to be studied whether the variable expression levels of imprinting genes are related with developmental potential of individual SCNT blastocysts.

Finally, in the case of EMGs, quite a few genes showed faulty expression as inferred by a tendency toward greater expression in the SCNT groups (Table 3). The differentially expressed EMGs were mostly histone deacetylases (*HDAC3*, *HDAC 4*, *HDAC7*, and *HDAC9*) and histone demethylases (*JMJD5*, *JMJD6*, *KDM1B*, *KDM3A*, *KDM5C*, and *KDM6A*). Notably, *HDAC3*, *HDAC9*, *RBBP8*, and *KDM5C* were detected in both cSCNT and fSCNT groups. Additionally, *TET3*, which hydroxylates existing 5-methylcytosine (Branco *et al.* 2012), was highly expressed in all of the SCNT blastocysts except for cSCNT. *EZH1* and *EZH2*, essential members of polycomb repressive complex 2 with H3K27 methylating activity (Margueron and Reinberg 2011), were also abnormally expressed in either of the two SCNT groups. Figure 4B shows differential expressions of representative genes of each category among blastocysts of different groups. Although some genes (*TUBB3*, *FOXJ3*, *EZH1*, and *EZH2*) were expressed at a relatively constant level within a group, others (*NANOG*, *CDX2*, *GCM1*, and *GATA4*) were expressed in a highly heterogenic fashion among different blastocysts. Observing such an expression heterogeneity within a group is the benefit of the RNA-seq analysis of single embryos.

## Discussion

Successful SCNT partly relies on the types of donor cells. It is well known that somatic cells from various tissues have distinct genetic and epigenetic backgrounds, and these differences affect reprogramming processes occurring over the genomes in SCNT embryos and their cloning efficiencies. As donor cells, both cumulus cells and ear-skin fibroblasts are the most popular in SCNT because they are readily available and well maintained under *in vitro* culture. In SCNT, cumulus cells and fibroblasts have long been comparable donor cells, and cloning efficiencies with these donor cells have been reported in goats and rabbits (Campbell *et al.* 2007; Tian *et al.* 2012) as well as in bovines (Liu *et al.* 2013). Consistently, several studies have observed that, among adult cell types for SCNT, adult skin fibroblasts and cumulus cells are better choices due to their greater cloning efficiency and lesser anomalies in the cloned animals (Kato *et al.* 1998, 2000; Pandey *et al.* 2010; Wells *et al.* 1999). Our recent quantitative analysis of gene expression profile using multiplex RT-PCR for reprogramming-related genes showed the same conclusion: cSCNT blastocysts are better in gene expression profile than fSCNT blastocysts (Kwon *et al.* 2015a,b). Thus, our RNA-seq result conforms to the widely accepted notion that cSCNT embryos are superior to fSCNT embryos, which in turn supports the reliability of our results from RNA-seq experiments equipped with the pico-profiling method.

In this study, we compared the transcriptome profiles between IVF and SCNT blastocysts. Our data lacked control transcriptome data from, for example, parthenogenetic blastocyst, other cleavage-stage embryo, and the donor cell, each of which could serve a meaningful reference depending on the experimental purpose. Our reasoning is, because the blastocyst stage is the furthest point that early embryos can reach *in vitro*, to what extent reprogramming has occurred could be judged by thorough scrutinization of SCNT blastocysts for their transcriptomic similarity to IVF standard, as we just focused the exploration of the result of genome reprogramming, instead of the reprogramming progress itself. In compensation for the simple comparison, we analyzed single SCNT blastocysts, which allowed us to detect a significant transcriptomic variation among them. The DEGs we identified in this way were largely commonly present in the blastocysts of the cSCNT and fSCNT groups, and this consistent appearance lays a great potential on these DEGs as practical expression markers that help us determine the efficiency of reprogramming in SCNT embryos.

The analysis of single embryos allowed us to understand to what extent the transcriptomic similarity and variation run among different blastocysts in each SCNT group. For example, we recognized that aberrant expressions of TEGs were not centered onto particular SCNT blastocysts but were instead largely spread among the SCNT blastocysts (Figure 4B); *CDX2* was significantly underrepresented in fSCNT1, *GCM1* in cSCNT3, and *EPCAM* in cSCNT2. *NANOG* and *GATA4* genes are other examples of heterogenic expression among individual blastocysts of the same groups. These results came from studying single embryos and might have not been surfaced if we had dealt with average gene expression levels of pooled blastocyst samples. Given the homogeneity in the donor cell population, these transcriptomic variations might reflect differential or stochastic reprogramming among the blastocysts. Although the number of blastocyst samples and their transcriptome data we obtained were not enough to draw out any featured pattern from the corresponding SCNT group, we could envisage how far the transcriptomic deviation goes in each SCNT group and in what proportion the blastocysts with abnormal transcriptome comprise certain SCNT group.

In fSCNT blastocysts, we found several reprogramming-resistant regions where bundles of adjacent genes were coordinately dysregulated (Figure 3). These genomic patches were for the most part singular to fSCNT embryos, either not found or only scarcely distinguishable in cSCNT blastocysts as reprogramming-resistant regions. Therefore, our result indicates that reprogramming-resistant regions in SCNT embryos tend to be predetermined to a degree by the type of donor cells.

The epigenomic state of early embryos is set and largely governed by a group of genes (EMGs, as named previously) that play roles in reading, writing, and erasing the epigenetic marks of chromatin. These EMGs are of particular importance for SCNT embryos because they are supposed to undergo substantial reprogramming process for development. Compared with other categories of genes, such as PGs (10%) and DRGs (10%), a relatively high proportion (26%) of EMGs was differentially expressed in the cSCNT and fSCNT groups (Table 3). This relatively large number of differentially expressed EMGs and their presumably high enzyme redundancy make it difficult to anticipate the molecular consequences of the unbalanced expressions of EMGs on chromatin state and reprogramming. Moreover, since the EMG products, by their intrinsic nature, act genome-wide, their faulty expressions could give rise to pleiotropic influences over the entire genome; therefore, once the errors are inscribed by unbalanced epigenetic systems, they are seldom fixed. All of these results highlight the importance of correct and balanced expression of the EMGs, which may guarantee proper epigenetic reprogramming and survival of SCNT embryos.

Global epigenetic state, or epigenome, of a certain cell is determined by complicated three-dimensional interactions of epigenetic modifiers and is the outcome of a balanced expression of sets of the “writers” and “erasers.” Our result showed that, as to the expression of EMGs, fSCNT blastocysts were faultier than cSCNT blastocysts (9/89 *vs.* 14/89 in Table 3). What consequences this difference may bring in to the epigenome is completely unknown because the cause-and-effect relations in generating an epigenome among the modifiers are inconceivably complex. A simple speculation could be that fSCNT blastocysts would maintain more erroneous (*i.e.*, less reprogrammed) epigenetic states than cSCNT and thus retain a lower developmental potential.

The IVF blastocysts used here were all expressed Y-linked genes (*ZRSR2Y*, *USP9Y*, *UTY*, *DDX3Y*, *PRAME*, and *EIF2S3Y*), whereas SCNT blastocysts were female. Therefore, it raises the possibility that our DEGs are the result of sexual dimorphism between male IVF and female SCNT blastocysts (Bermejo-Alvarez *et al.* 2011; Gutierrez-Adan *et al.* 2006; Kobayashi *et al.* 2006). We observed from heatmap display over X chromosomes that, although X-linked genes appeared up-regulated in general, their fold changes were mostly lower than 1.6 and even those >1.6 were statistically insignificant (*P* < 0.05; data not shown), as previously noted (Bermejo-Alvarez *et al.* 2010). A gene expression study by DNA microarray comparison of bovine IVF blastocysts showed that many X-linked-expressed transcripts were up-regulated in females and reported around 50 confident DEGs (Bermejo-Alvarez *et al.* 2010). However, when we compared those DEGs with our 78 SCNT-high DEGs, only six DEGs overlapped: three on the X chromosome (*SAT1*, *SLITRK2*, and *XIST*) and three on other autosomes (*ALDOC*, *HSPA1A*, and *LOC618696*). Moreover, although that study only found two up-regulated genes in male blastocysts, ours detected 56 overexpressed DEGs in males, which accounts for far more than the male−female difference, supporting that our DEGs are mostly derived from the IVF−SCNT difference. We could, therefore, say that the male−female difference was outweighed by the IVF−SCNT difference in the exploration of DEGs. It is unclear why such a difference in transcriptional sexual dimorphism exists between our result and the previous microarray data. This could be explained by female SCNT blastocysts expressing far fewer X-linked genes than female IVF blastocysts because the former somehow more tightly sustains an inactive X state or fails to reactivate an inactive X chromosome during preimplantation development. A relevant answer could be taken from a transcriptomic comparison between female IVF and female SCNT embryos, which we are currently researching.

As far as we know, this is the first report about RNA-seq on single-bovine SCNT blastocysts. Our efforts to elucidate the reprogramming inefficiency of SCNT embryos by gene expression profiling will help to understand the stochastic reprogramming events to achieve better cloning efficiency. The identified DEGs and several stretches of genomic regions displaying a reprogramming resistant nature are supposed to serve as genetic markers in future study which aims to find a way to promote reprogramming process in SCNT embryos.

## 

## Supplementary Material

Supporting Information
