# Supplementary material for: Transcriptomic Features of Bovine Blastocysts Derived by Somatic Cell Nuclear Transfer
Source: G3 (Bethesda). 2015 Sep 3;5(12):2527–38. doi: 10.1534/g3.115.020016 (PMC4683625; doi:10.1534/g3.115.020016)
Supplement: Supporting Information [file supp_5_12_2527__index.html]

Transcriptomic Features of Bovine Blastocysts Derived by Somatic Cell Nuclear Transfer — Supporting Information 

# Transcriptomic Features of Bovine Blastocysts Derived by Somatic Cell Nuclear Transfer

## Supporting Information for Min *et al.*, 2015

**Files in this Data Supplement:**

- Supporting Information - Figures S1-S3 and Files S1-S2 (PDF, 1 MB)
- Figure S1 - Differentially expressed genes (fold change of >2 and p < 0.05) between cSCNT and fSCNT blastocysts. (PDF, 565 KB)
- Figure S2 - Individual cSCNT expression profiles against IVF mean at the zinc-finger protein gene cluster. (PDF, 205 KB)
- Figure S3 - Expression profiles of imprinting genes. Heatmap displays relative expressions in individual blastocysts against IVF mean. (PDF, 475 KB)
- File S1 - Normalized expression profiles of total genes. (.xlsx, 2 MB)
- File S2 - Expression profiles of pluripotency (PG), trophetodem (TEG), development (DRG), and epigenetic modifying (EMG) genes. (.xlsx, 41 KB)
