# Supplementary material for: Transcriptomic Features of Bovine Blastocysts Derived by Somatic Cell Nuclear Transfer
Source: G3 (Bethesda). 2015 Sep 3;5(12):2527–38. doi: 10.1534/g3.115.020016 (PMC4683625; doi:10.1534/g3.115.020016)
Supplement: Supporting Information [file supp_g3.115.020016_020016SI.pdf]

G3: Genes | Genomes | Genetics

Transcriptomic features of bovine blastocysts derived by somatic cell nuclear transfer

Byungkuk Min[1,2], Sunwha Cho[1,2], Jung Sun Park[1], Yun-Gyeong Lee[3,4], Namshin Kim[3,4], and Yong-Kook Kang[1,2]*

[1] Development and Differentiation Research Center, KRIBB, 125 Gwahak-ro, Yuseong-gu, Daejeon, 305-806, South Korea

[2] Department of Functional Genomics, University of Science and Technology (UST), 217 Gajeong-ro Yuseong-gu, Daejeon, 305-350, South Korea

[3] Department of Bioinformatics, University of Science and Technology (UST), 217 Gajeong-ro Yuseong-gu, Daejeon, 305-350, South Korea

[4] Korean Bioinformation Center, KRIBB, 125 Gwahak-ro, Yuseong-gu, Daejeon, 305-806, South Korea

* To whom correspondence should be addressed: Yong-Kook Kang, Development and Differentiation Research Center, KRIBB, 125 Gwahak-ro, Yuseong-gu, Daejeon 305-806, South Korea, Tel.: +82 42 860 4427; Fax.: +82 42 860 4608; E-mail: ykkang@kribb.re.kr

DOI: 10.1534/g3.115.020016

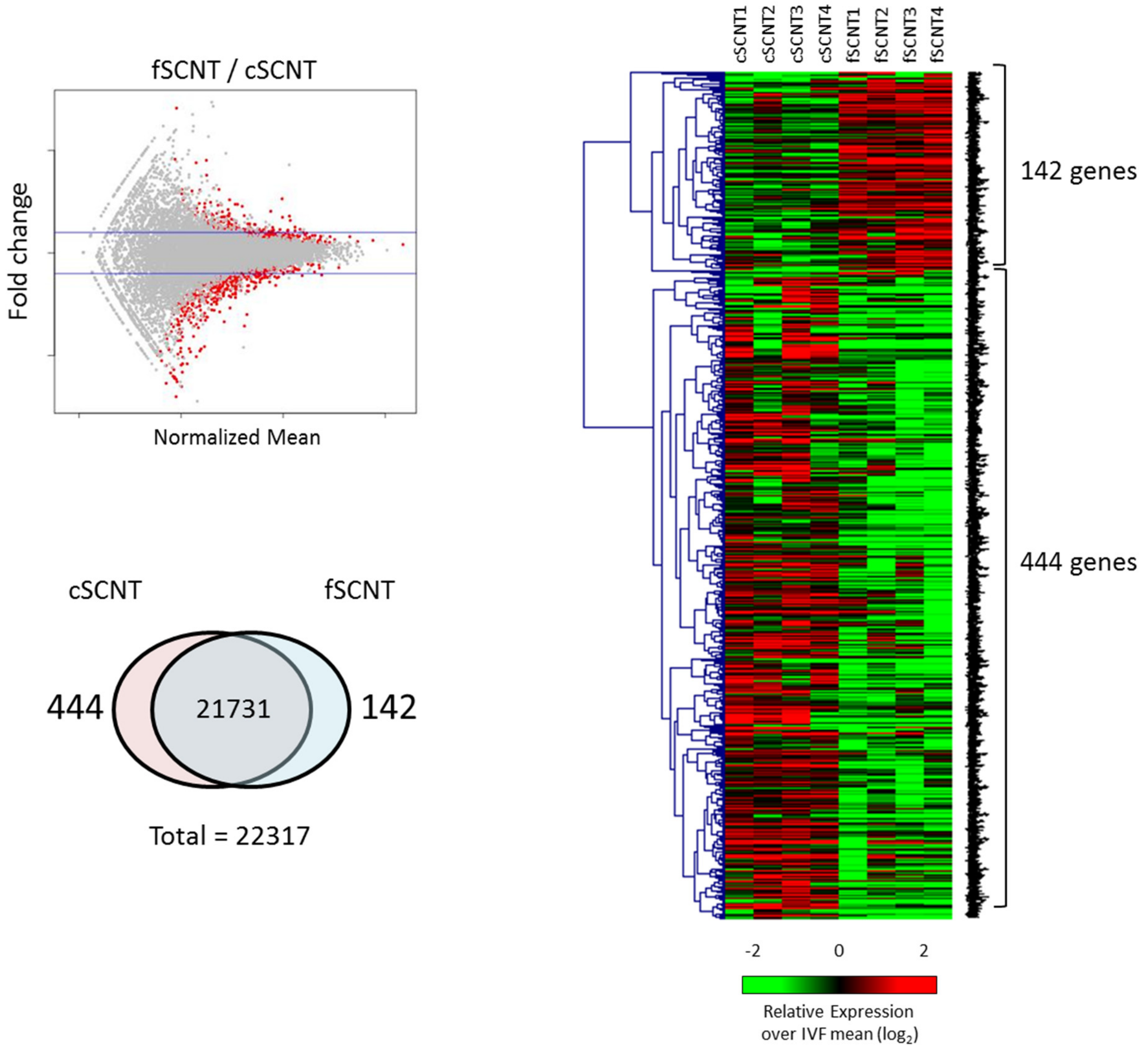

Figure S1 Differentially expressed genes (fold change of >2 and $p < 0.05$) between cSCNT and fSCNT blastocysts. A, DESeq analysis. B, Venn diagrams. C, Heatmap

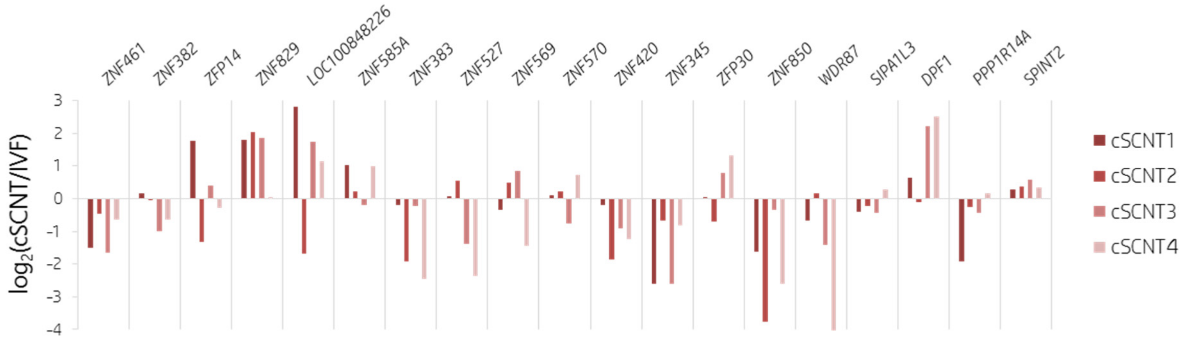

Figure S2 Individual cSCNT expression profiles against IVF mean at the zinc-finger protein gene cluster.

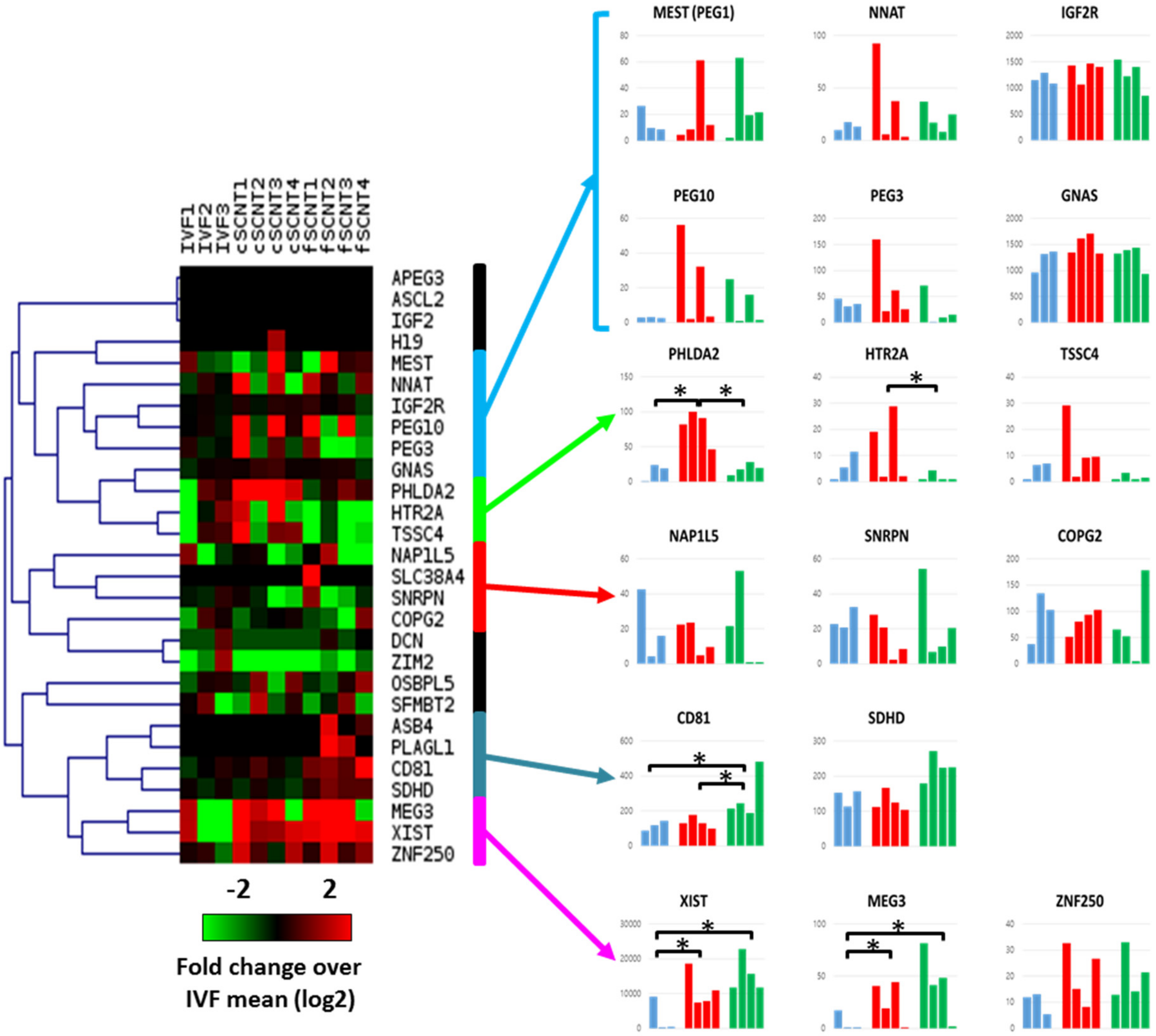

Figure S3 Expression profiles of imprinting genes. Heatmap displays relative expressions in individual blastocysts against IVF mean. Detailed expression differences of selected genes among individual blastocysts are shown as bar charts on the right.

Files S1-S2

Available for download at www.g3journal.org/lookup/suppl/doi:10.1534/g3.115.020016/-/DC1

File S1 Normalized expression profiles of total genes

File S2 Expression profiles of pluripotency (PG), trophetodem (TEG), development (DRG), and epigenetic modifying (EMG) genes
