# Supplementary material for: Transcriptomic Features of Bovine Blastocysts Derived by Somatic Cell Nuclear Transfer
Source: G3 (Bethesda). 2015 Sep 3;5(12):2527–38. doi: 10.1534/g3.115.020016 (PMC4683625; doi:10.1534/g3.115.020016)
Supplement: Supporting Information [file supp_g3.115.020016_FigureS2.pdf]

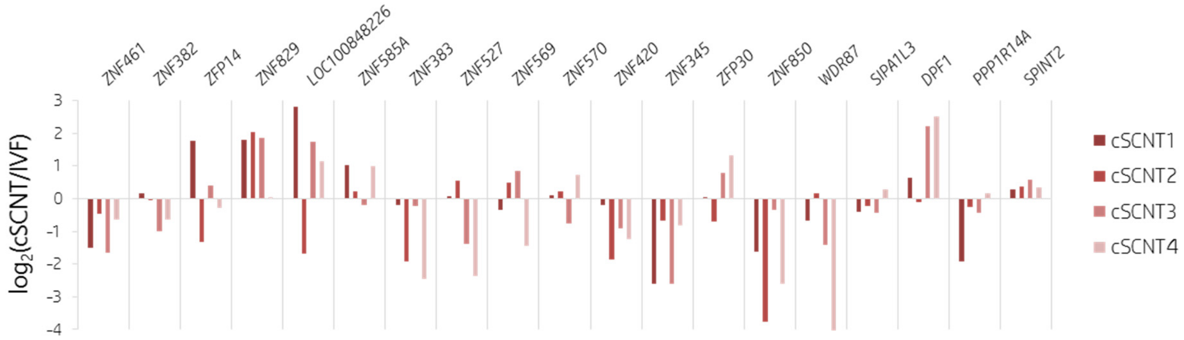

Figure S2 Individual cSCNT expression profiles against IVF mean at the zinc-finger protein gene cluster.
