# Supplementary material for: Transcriptomic Features of Bovine Blastocysts Derived by Somatic Cell Nuclear Transfer
Source: G3 (Bethesda). 2015 Sep 3;5(12):2527–38. doi: 10.1534/g3.115.020016 (PMC4683625; doi:10.1534/g3.115.020016)
Supplement: Supporting Information [file supp_g3.115.020016_FigureS3.pdf]

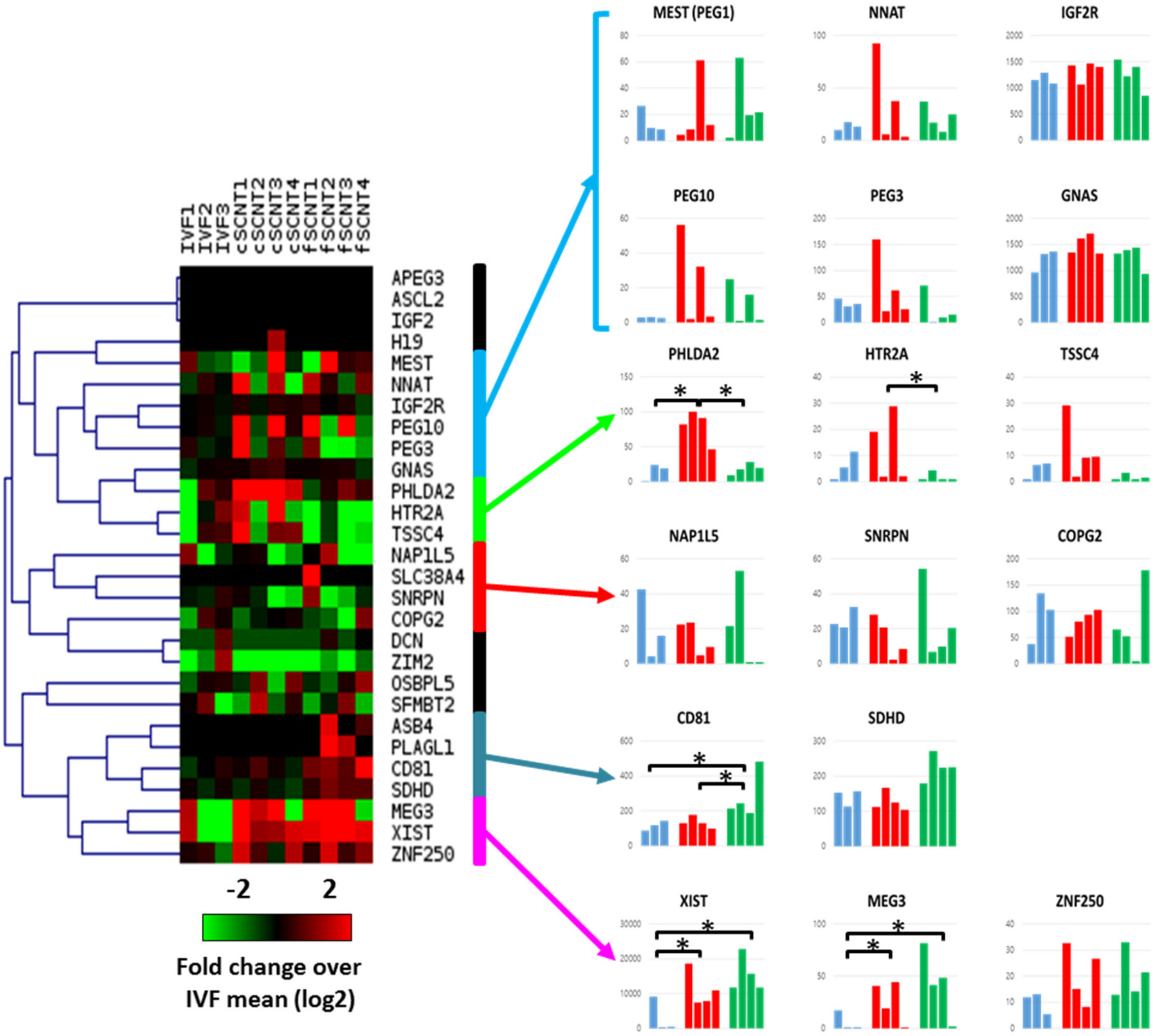

Figure S3 Expression profiles of imprinting genes. Heatmap displays relative expressions in individual blastocysts against IVF mean. Detailed expression differences of selected genes among individual blastocysts are shown as bar charts on the right.
